# Retraction Note to: First report of the Phe1534Cys kdr mutation in natural populations of *Aedes albopictus* from Brazil

**DOI:** 10.1186/s13071-022-05382-9

**Published:** 2022-07-22

**Authors:** Oscar Alexander Aguirre-Obando, Ademir Jesus Martins, Mário Antônio Navarro-Silva

**Affiliations:** 1 Laboratório de Entomologia Médica e Veterinária, Departamento de Zoologia, Universidade Federal do Paraná, Setor de Ciências Biológicas, PO Box 19020, Curitiba, Paraná 81531-980 Brazil; 2 Laboratório de Fisiologia e Controle de Artrópodes Vetores, Instituto Oswaldo Cruz-FIOCRUZ, Av. Brasil 4365, PO Box 2104-900, Rio de Janeiro, RJ Brazil; 3 Instituto Nacional de Ciência e Tecnologia em Entomologia Molecular, Rio de Janeiro, RJ Brazil

## Retraction to: Parasites & Vectors (2017) 10:160 http://doi.org/10.1186/s13071-017-2089-5

The authors have retracted this article because of evidence of cross-contamination in the samples used.

In the above-mentioned paper, we performed individual DNA extractions of *Ae. albopictus* samples collected in three Brazilian states (Paraná, Mato Grosso and Rondônia). The DNAs were pooled to each specific locality, with which we amplified two fragments of the voltage-gated sodium channel gene (*Na*_*V*_), corresponding to the segments IIS6 and IIIS6 of this channel. The Na_V_ protein is the target of pyrethroid insecticides, and mutations conferring resistance to the knockdown effect are known as *kdr* mutations. We used primers previously designed for *Ae. aegypti* to amplify both IIS6 and IIIS6 segments, as their annealing sequences were considered identical in *Ae. albopictus*. The PCR products were cloned and sequenced in order to disclose the molecular diversity of these segments and in search of *kdr* mutations, as previously observed in these segments.

Among our obtained sequences, we observed two haplotypes in the IIS6 segment, highly similar to the few other homologous sequences of *Ae. albopictus* available at that time. Especially in this segment, there is a highly variable intron, with remarkedly differences in the alignment of *Ae. albopictus* and *Ae. aegypti* IIS6 sequences. Our obtained haplotypes well-evidenced this. In the IIIS6 segment, we also found two haplotypes (KX371864 and KX371865, GenBank accession number). At that time in the GenBank, there were few IIIS6 homologous sequences of *Ae. albopictus* available, and none of them covered all our sequenced fragment, especially in the intron. We reported that these two IIIS6 haplotypes were very similar to the *Ae. aegypti* previously described, including the presence of the *kdr* mutation F1534C.

Based on these results, we performed allele-specific PCR (AS-PCR) to genotype individual samples to determine the *kdr* allelic frequencies, identified between 0 and 10%, and only found in heterozygosis. We concluded that the F1534C *kdr* mutation (previously evidenced in *Ae. aegypti* from several countries, including Brazil) was for the first time found in Brazilian *Ae. albopictus* populations, yet under low frequencies.

Since this publication, there have been several submissions of additional *Ae. albopictus Na*_*V*_ from worldwide in the GenBank. We also continued our studies and found several other haplotypes in the *Na*_*V*_ gene samples in Brazil, using next-generation sequencing approaches (unpublished observations). When we align our published IIIS6 haplotypes (KX371864 and KX371865), we observe that they are more similar to *Ae. aegypti* sequences, instead of with *Ae. albopictus*. This suggested that we might have had contamination in our samples. In this case, our IIIS6 primers would be more efficient in amplifying the *Ae. aegypti* DNA contaminant. Then, we returned to some DNA samples that presented one of those haplotypes and amplified and sequenced the *COI* gene fragment. This analysis evidenced *Ae. aegypti* DNA cross-contamination in some of our *Ae. albopictus* DNA samples.

The source of contamination is unclear. We collected eggs in the field, from which larvae hatched in the laboratory and were raised until adulthood, when they were then separated in *Ae. aegypti* and *Ae. albopictus*. In total, only 3% were *Ae. albopictus* (Table 1, in the original publication). Therefore, it is possible that a fragment of *Ae. aegypti* body might have been attached to some of the *Ae. albopictus* samples, enough to have DNA extracted and amplified.

Whatever be the contamination source, we are now sure that the IIIS6 haplotypes we published are from *Ae. aegypti* and not from *Ae. albopictus*. Consequently, we cannot be certain about the occurrence of the *kdr* mutation in those samples and if the AS-PCR results reflect its frequency in the evaluated *Ae. albopictus* populations. We requested that the sequences of the IIIS6 haplotypes be removed from the GenBank.

We are aware that this publication was cited in several subsequent studies and revisions (including ours), and we are deeply sorry about this mistake. At least we found the error, and we hope that this case may serve as an additional alert about the consequences of cross-contamination in the molecular biology bench-work practices. We will continue our studies with vector molecular biology and hopefully bring more light to this field of knowledge, compensating for any confusion we might have caused with this publication. All authors agree with this retraction.

